# Harnessing the Potential of Wheat-Pea Species Mixtures: Evaluation of Multifunctional Performance and Wheat Diversity

**DOI:** 10.3389/fpls.2022.846237

**Published:** 2022-03-25

**Authors:** Johannes Timaeus, Odette Denise Weedon, Maria Renate Finckh

**Affiliations:** Department of Ecological Plant Protection, Faculty of Organic Agricultural Sciences, University of Kassel, Witzenhausen, Germany

**Keywords:** species mixtures, intercropping, diversification, heterogeneous population, multifunctional agriculture, yield gain, composite cross

## Abstract

Species mixtures and heterogeneous crop populations are two promising approaches for diversified ecological cropping systems with increased resilience and reduced dependency on external inputs. Inter- and intraspecific diversity were evaluated in combination using 15 wheat (*Triticum aestivum*) entries, including line cultivars and heterogeneous populations (HPs), from central Europe and Hungary and one winter pea cultivar under organic conditions. Monocultures and wheat mixtures were evaluated multi-functionally for yield, quality, land use efficiency, crop protection, and wheat entry traits. Mixtures increased cereal grain quality, weed suppression, resource use efficiency, yield gain, and reduced lodging. Effects were stronger in 2018/19, which were characterized by dry and nutrient-poor conditions than in 2019/20 when nutrient levels were higher. Wheat entries varied considerably in protein content and yield in both mixtures and monocultures. Under higher nutrient availability, entry-based variation was reduced in both systems, and peas were suppressed. Because of low disease pressure, the wheat entries varied little in terms of disease protection services, and mixture effects on the disease were low. The multi-criteria framework identified stability of yield, yield gains, and quality under high environmental variability of mixtures as clear agronomic advantages with HPs being considerably more stable than line cultivars. Some line cultivars outperformed the HPs in either protein content or yield across environments but not both simultaneously. Trait analysis revealed a possible link between harvest index and reduced competition in mixtures, which can increase yield performance in specific line cultivars. System cultivar interactions were generally very low and highly dependent on environmental conditions. We conclude that while cultivar breeding for mixtures can be successful in monocultures, high environmental variation highlights the necessity of evaluating cultivars in mixtures. In addition, use of intraspecific diversity within interspecific mixed cropping systems can be a valuable addition to further improve mixture performance and its stability under increasing environmental stresses due to climate change.

## Introduction

High-input single species cropping systems are very vulnerable to unpredictable climatic conditions that are the result of climate change (IPCC, 2021, Chapt. 11). Additionally, they rely on fossil fuel-based nitrogen fertilizers and plant protection chemicals for reliable productivity. Nitrogen fertilizer production requires 65–100 MJ per kg with associated emissions of 2.1–5.5 kg CO_2_ equivalents per kg (Jensen et al., 2020). There is an urgent need for highly resilient and resource-efficient cropping systems that contribute simultaneously to climate change adaptation and mitigation. Intra- and interspecific diversification of cropping systems has been identified as one of the important building blocks of such agricultural systems (Østergård et al., 2009; Finckh et al., 2021).

Intraspecific diversity enhances resilience against biotic and abiotic stress and can be achieved through evolutionary breeding approaches resulting in HPs (Suneson, 1956; Finckh, 2008; Döring et al., 2011). HPs are highly adaptive to environmental stress and provide higher yield stability than genetically homogenous line cultivars in wheat (Brumlop et al., 2017, 2019; Weedon and Finckh, 2019, 2021). In recognition of the valuable contribution of intraspecific diversity to crop resilience, the new EU Organic Regulation 2018/848 that will come into force in 2022 will provide a legal framework for HPs, enabling further mainstreaming.^1^

Intraspecific diversity in cropping systems can be further enhanced by adding interspecific diversity. A key lever to harness interspecific diversity in agriculture is to use the complementarity in nitrogen acquisition strategies of cereals and legumes (Bedoussac et al., 2015). A recent global study estimated that intercropping cereals and legumes could decrease the required fertilizer globally by 26% compared to sole crops (Jensen et al., 2020). In addition, cereal-legume species mixtures in arable cropping systems provide services, such as improved crop quality, weed suppression, land-use efficiency (Bedoussac et al., 2015), crop health (Finckh et al., 2000; Boudreau, 2013; Finckh and Wolfe, 2015), and lodging resistance (Kontturi et al., 2011; Podgórska-Lesiak and Sobkowicz, 2013). Species mixtures also provide ecosystem services, such as soil (Stefan et al., 2021) and water conservation (Yin et al., 2020).

A major effect of plant species diversity on natural ecosystems and cultivated grasslands is increase in ecosystem multifunctionality (Hector and Bagchi, 2007; Gamfeldt et al., 2008; Isbell et al., 2011, 2017; Suter et al., 2021). Consequently, taking a multifunctional perspective on arable cropping systems is needed to fully appraise cropping system diversity (Huang et al., 2015; Schmidtke, 2021). Studies that assess genotype effects of species mixtures on yield, quality, resource efficiency, and crop protection from a multifunctional perspective are missing, and many studies are only focused on some aspects of cropping system performance.

Species mixtures of wheat (*Triticum aestivum* L.) and pea (*Pisum sativum* L.) are touted as a model species mixture (Pelzer et al., 2012; Mamine and Farès, 2020), as these two species are complementary for many needs and, in combination, may help in mitigation of climate change-related challenges. Wheat protein content and baking quality strongly depend on timely plant-available soil nitrogen (Wieser and Seilmeier, 1998; Xue et al., 2016). However, high-input fertilization practices to improve wheat quality provide nutrients throughout the season, which are only partially utilized by the crop, often resulting in nutrient leaching into the environment (Häußermann et al., 2019). Nitrogen (N) uptake by winter wheat in the fall is minimal; however, sufficient availability of N during grain filling is critical to achieve good baking quality (Xue et al., 2016). Multiple mechanisms can contribute to improved wheat grain quality in species mixtures, and the most often cited is reduced competition for nitrogen in mixtures compared to wheat monocultures (Bedoussac et al., 2015; Stomph et al., 2020), but other mechanisms, such as transfer of nitrogen from legumes to non-legumes, have also been discussed for pasture ecosystems (Thilakarathna et al., 2016). Winter peas with determined growth, flower and mature earlier than most winter wheat cultivars (Bioland, 2021). Biological nitrogen fixation of legumes ceases after flowering, and N is released from nodules. If this coincides with N requirement during grain filling of wheat, grain protein content should be improved. This may interact with water use efficiency in mixtures that can be higher than in monocultures because of several mechanisms, such as change in evapotranspiration, hydraulic lift, and Spatio-temporal differentiation of water use. For example, Daryanto et al. (2020) found that moisture in deeper soil levels can be reduced while increased in shallower soil levels in mixtures compared to monocultures. Peas can shade the ground in between wheat plants, which, besides suppressing weeds, help reduce soil temperature and evaporation, potentially mitigating soil drought conditions that hamper soil mineralization processes, and reduce nitrogen availability. On the other hand, pea monocropping systems face a range of challenges, such as lodging, pests, and diseases, and high weed pressure, causing strong yield fluctuations (Watson et al., 2017), which can potentially be mitigated by mixed cropping. In contrast, in single-species cropping systems, these challenges can only be controlled by increased external inputs hindering climate change mitigation. Most empirical research studies on cultivar effects in legume-cereal mixtures so far have focused on legume cultivars, such as pea cultivars in mixtures with cereals (Hauggaard-Nielsen and Jensen, 2001; Annicchiarico et al., 2017, 2019; Baxevanos et al., 2017; Haug et al., 2021). Broader and systematic evaluation of cereal cultivar effects is missing so far. Considering the value of intraspecific diversity, it is of interest to assess both homogeneous line cultivars and HPs in this context, as suggested by Saxena et al. (2018) and Annicchiarico et al. (2019) for legumes. Addition of HPs can contribute to improve species mixture performance and integrate biological diversity at multiple levels of cropping systems, including diversity at the inter- and intraspecific levels.

This study addresses two main research goals. First, an overall multifunctional performance evaluation of wheat-pea species mixtures is conducted by comparing them to pea and wheat monocultures for yield, crop quality, resource efficiency, and crop protection services. Second, wheat cultivars, including line cultivars and HPs, are evaluated addressing three secondary aims: (a) study the magnitude of cultivar effects and system-cultivar interactions to evaluate the need to specifically breed for species mixtures, (b) compare the performance and stability of line cultivars and HPs, (c) identify candidate traits, such as phenological, and yield traits, such as harvest index, that explain system-cultivar interactions and performance in mixtures. The secondary aims are crucial to improve breeding for species mixtures.

In the experiments presented here, sowing densities were partially additive (70% of wheat pure stands and 50% of pea pure stands), with an explicit aim to enhance wheat performance especially with respect to baking quality. Total density (pea + wheat) in mixtures was therefore 83% (290 seeds/m^2^) of wheat pure stand (350 seeds/m^2^) and 322% of pea pure stand (90 seeds/m^2^). Additive designs make it difficult to distinguish density from mixture effects, in contrast to replacement designs where total densities are held constant (Harper, 1977; Bybee-Finley and Ryan, 2018). However, additive designs are used more often in practice than replacement designs (FiBL, 2017). Therefore, our main research question addresses mixture effects under realistic farming conditions and wheat cultivar effects on mixture performance.

## Materials and Methods

### Study Site and Design

Experiments were conducted in 2017/18, 2018/19, and 2019/20 at the University of Kassel Research Station in Neu-Eichenberg (51°22′24.7″ N and 9°54′12.5″ E, 247 m asl). The soil is classified as Haplic Luvisol with 76 soil points according to the German soil classification system (0–100). Mean annual temperature from 2000 to 2020 was 9.1°C, and the mean annual precipitation was 626 mm. The site has been managed organically since 1982 without the addition of synthetic fertilizers or pesticides. In 2019/20, 20 t ha^–1^ vetch-rye silage was added to the field as a routine nutrient amendment. Weeds were controlled by harrowing and hoeing at tillering. A split-plot design with four replicate blocks with mixtures/monocultures as main plots and randomized cultivar plots nested within the main plot for each block was used. Plot size was 13 × 1.5 m^2^ with five rows at 28 cm distance. Wheat and pea monocultures were sown with 350 and 90 seeds m^–2^, respectively. Sowing rates in mixtures were 70% for wheat and 50% for pea to increase protein values in wheat. Weather data were recorded by the weather station located in the experimental site.

Soil N levels were 17, 25, and 42 kg N ha ^–1^ at a depth of 0–60 cm in February 2018, 2019, and 2020, respectively. In June 2019 and 2020 (BBCH of wheat 70–80), nitrogen levels were 11 and 22 kg, respectively. Data for 2018 could not be taken because of extreme drought.

**FIGURE 1:**
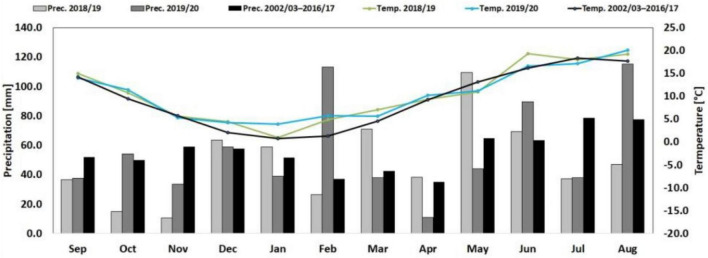
Monthly mean precipitation and temperature for two experimental seasons and the long-term means (2002/2003–2016/17). Weather data were obtained from the weather station located in the research station.

The mean temperature in the 2017/18 season was 10.5°C; the total precipitation was 471 mm and deviated considerably from the long-term annual mean. Black frost, in February 2018 killed nearly all winter peas, was followed by extremely dry conditions throughout critical developmental phases of wheat. These dry conditions persisted until late November 2018, also affecting the second season at sowing (Figure 1). In 2018/19 and 2019/20, mean temperatures were 10.4°C with warm winter but less extreme summer. The total annual precipitation of 583 mm in 2018/19 did not compensate for the 2017/18 drought, but it was adequate for wheat growth. In May 2019, torrential rain of 70 mm in 3 h resulted in heavy lodging of pea monocultures. In 2019/20, total precipitation was similar to the long-term average of 671 mm.

### Plant Material

In 2017/18, the winter pea cultivar “Dexter” (white flowers, *determinate* short stature) was sown but killed in February 2018 by black frost. Therefore, in 2018/19 and 2019/20, the more frost-tolerant winter pea cultivar “Fresnel” was chosen. “Fresnel” is a French winter pea cultivar with determinate growth, short stature, and early maturation suited for mixtures with barley (Petersen, 2021).

The wheat entries consisted of line cultivars and HPs. The year 2018 was extremely hot and dry. Therefore, an HP (H-HP) and four cultivars from Hungary (H-lines) were included in 2018/19 and 2019/20 to include more adapted materials to summer heat and increase the trait variation of wheat (Table 1). The line cultivars “Butaro” and “Wiwa” were bred organically by Dottenfelderhof and Getreidezüchtung Peter Kunz, respectively (Bioverita, 2020). “Achat” and “Capo,” both bred by Probstdorfer Saatzucht, Austria, are baking cultivars popular in organic farming and have relatively good foliar health (Naturland, 2018). The Hungarian line cultivars “Nemere,” “Toborzo,” “Kolompos,” and “Karizma” were bred by the Agricultural Research Center of the Hungarian Academy of Science in Martonvásár.

**TABLE 1:** Wheat entries used, grouped by type (HP, heterogeneous population; Line, line cultivar) and origin (C, central Europe; H, Hungary, K, Kassel University; D, Dottenfelder Hof).

Group	Entries (origin)
C-Lines	Achat (DE), Butaro (DE), Capo (AU), Wiwa (DE),
H-Lines	Karizma, Kolompos, Nemere, Toborzo, (HU)
K-HPs^2^	OYQII, OQII, BSFI, BSFII (2001 in United Kingdom, since F_5_ at Univ. of Kassel, used generations ≥ F_16_)
D-HPs	Brandex, Liocharls, (DE)
H-HPs	Elit CCP (HU)

The HPs from the University of Kassel (K-HP, Table 1) evolved from HPs created in 2001 by the Organic Research Centre and the John Innes Institute in the United Kingdom (Döring et al., 2015). “OYQI” and “OYQII” are the result of crossing 8 high-yielding (Y) × 11 baking quality (Q) parents, plus all 19 parents crossed with the cultivar “Bezostaya,” while “OQI” and “OQII” resulted from a half-diallel cross of 12 baking quality cultivars. Since 2005 (F_5_), these HPs have been maintained under organic (O) conditions as parallel non-mixing (I, II) populations at the University of Kassel without conscious selection, apart from the removal of plants taller than 130 cm during the first 3 years (Brumlop et al., 2019). “OYQII” is registered in Germany under the name “EQuality” (OSS, 2020). In F_8_, a pooled seed of “OYQI” and “OYQII” was sown broadcast and maintained without mechanical weed control in two non-mixing populations since F_9_ (“BSFI,” “BSFII”) (Döring et al., 2015; Brumlop et al., 2017, 2019). “Brandex” and “Liocharls” are recently released German organic baking quality HPs bred by Dottenfelderhof (D-HP; Spieß and Vollenweider, 2016), and “Elit CCP” is a Hungarian HP (H-HP) made of 7 high-yielding and high-quality Hungarian cultivars for which F_4_ was available (Costanzo and Bàrberi, 2016).

### Data Collection

All assessment dates are summarized in Supplementary Table 1. Plant emergence was determined along three row sections of 0.5 m in three inner rows of each plot to avoid edge effects (BBCH 10). BBCH growth stages (Lancashire et al., 1991) were assessed at each field visit and every second day starting at the end of booting to determine heading dates. Weed cover (%) was estimated visually before booting (BBCH 20–25) six times per plot in an area of 0.1 m^2^ using a metal sampling frame. Lodging was estimated as percentage of the plot area (BBCH 70–80). At maturity, three 0.5-m rows (0.42 m^2^) were cut, and wheat and pea plants were separated. Total dry biomass, grain weight, and thousand-grain weight (TGW) were measured, and the number of ear-bearing tillers was counted.

Non-green leaf area (NGLA, in %) due to disease and senescence was assessed visually twice per season starting when diseases became relevant in early/mid-June, and from beginning/end of flowering to end of milk stage (BBCH 50–70). The two most important causes of NGLA were recorded. Foot diseases were assessed in year two and in three of 8 entries in early July (BBCH 70–80) (“Achat,” “Butaro,” “Capo,” “Kolompos,” “Nemere,” “Toborzo,” “Elit CCP,” and “OYQII”). Per plot, a total of 30 tillers were pulled out with the root crown from five to six places within the plot. The outer stem sheaths were removed and symptoms of *Fusarium* spp., *Oculimacula yallundae*, and *Ceratobasidium cereale* were identified on the stem base using a pictorial key (Bayer, 2013) and scored on a 0–3 scale (Bockmann, 1963), where 0 indicates a healthy stem, 1 (< 50%) and 2 ( > 50%) of the stem diameter show symptoms, and 3 indicates broken stems (*O. yallundae* only).

Grain yield was determined by combining harvesting and subsequent separation of pea and wheat grains and yields and adjusted to 14% moisture. A NIRS-based analysis of wheat grains for protein, gluten, and water content, sedimentation, and hectoliter weight was conducted with Foss Infratec 1,241 Grain Analyzer. NIRS results were used to categorize entries into wheat quality groups based on thresholds given for protein, where a protein content of < 10% is classified as fodder, 10–11% as second-class baking quality, and > 11% as first-class baking quality (Drangmeister, 2011).

### Data Processing and Statistical Analysis

The area under the curve for non-green leaf area (AUNGLA) was calculated as described by Shaner and Finney (1977) for AUDPC based on NGLA. Foot disease index (DIA) was calculated according to Bockmann (1963) for all three foot pathogens individually and combined as described in detail by Weedon and Finckh (2021). Harvest index (HI) was calculated as the ratio of grain to total biomass. Days to heading (DTHs) were calculated from the sowing date.

To assess cropping system performance, yield gain (YG) and land equivalent ratio (LER) were calculated. Yield gain (YG) quantifies differences in the yield of mixtures (*Y*_*mix_t_*_) compared to the expected mixture yield (*Y*_*mix_e_*_) calculated from pure stands adjusted by sowing densities (Li et al., 2020):


(1) $$YG=Y_{mix_{t}}-Y_{mix_{e}}$$

(2) $$Y_{mix_{e}}=\left(Y_{1mon}*D_{1}+Y_{2mon}*D_{2}\right)$$

where *D*_*n*_ is the relative density of species in mixture compared to monoculture.

LER quantifies the land area required in mixture relative to the area that would be required to obtain the same yield in pure stand, and is defined as the sum of yield ratios of both crop species in mixtures and pure stands (Mead and Willey, 1980):


(3) $$LER=\frac{Y_{1mix}}{Y_{1mon}}+\frac{Y_{2mix}}{Y_{2mon}}$$

where *Y*_*nmon*_ and *Y*_*n mix*_ are the yields of species *n* in monoculture and mixture, respectively.

Relative mixture effects (RMEs) were calculated based on response ratios (Hedges et al., 1999) for each individual plot:


(4) $$RME=\left(\frac{R_{mix}}{R_{mon}}-1\right)\times 100$$

where R_*n*_ refers to the response variable (yield, protein) in the respective system.

A mixed model approach was taken into account for the nested structure of the split-plot experiment (Piepho and Edmondson, 2018). Additionally, because of significant interactions between year and system for wheat and pea for experimental seasons 2018/19 and 2019/20 (Supplementary Table 4), experimental years were analyzed separately. For the response variable (R) for yield, protein, and diseases, two factorial models were built with system, entry, and replicate as fixed effects and main plot nested in replicate as a random effect:


R∼system*entry+replicate+1|replicate:mainplot

For YG and LER, models were constructed with wheat entry as fixed and replicate as a random effect:


R∼entry+1|replicate

If confidence intervals did not cross zero for yield gain and did not cross 1 for LER, mixture effects were judged as robust. For lodging data with many zero values, a glm with a Poisson family distribution was fitted.

The response ratio model for estimating relative mixture effect (RME) means and confidence intervals was specified as:


RME∼R+1|replicate

RMEs are present if estimated confidence intervals do not cross zero. Mixture effects could not be calculated for lodging data, as this would result in a denominator of zero. Response ratios were divided among four classes indicating multifunctionality: yield (pea and wheat, total yield), resource use efficiency (yield gain), crop protection (weeds, disease, lodging), and wheat grain quality (water, protein, and gluten content, hectoliter weight, and sedimentation).

The linear mixed models were complemented by a genotype main effect plus genotype-by-environment interaction analysis and biplots as a visual tool for analysis (GGE, Yan et al., 2007; Yan, 2014). GGE is a principal component analysis optimized for analysis of genotype suitability across environments and focuses on entry and entry-environment interaction effects. First, GGE was applied to assess entry association with cropping system and seasons defined as environments. In a further genotype by trait (GT) analysis, relationships between performance indicators (LER, protein, YG) and traits (HI, kernels per ear, tillers m^–2^, TGW, DTH) were analyzed to identify trait profiles of the entries.

All statistics were calculated using R (R. Core Team, 2020). Dplyr (Wickham et al., 2020b) was used for data aggregation and handling, and ggplot2 (Wickham et al., 2020a) and ggpubr (Kassambara, 2020) for plotting. Normality was assessed with histograms, and variance heteroscedasticity was tested by Levene’s test for model residuals and visual methods. The package lme4 (Bates et al., 2020) was used for mixed-effects models for absolute performance data, and nlme (Pinheiro et al., 2020) for mixed effect models with weighted variances to account for heteroscedasticity of response ratios. Estimated marginal means and confidence intervals (CIs) were calculated with the emmeans package (Lenth et al., 2020) followed by a *post-hoc* test with pairwise comparison and Holm correction. GLMs were constructed with base R. GGE and GT analyses were performed using the metan package (Olivoto and Lúcio, 2020).

## Results

In 2018/19, the mean pea emergence rate was 100% in the mixtures and 97% in the monocultures. Pea winter survival was 55 and 107 seedlings m^–2^in the mixtures and monocultures, respectively. Mean emergence rates of wheat were74 and 75% in the mixtures and monocultures, respectively. In 2019/20, mean pea emergence rates were 82% in the mixtures and 72% in the monocultures. Respective survival rates were 33 and 51 seedlings m^–2^in the mixtures and monocultures. Mean emergence rates of wheat were 86 and 80% in the mixtures and monocultures, respectively.

### Multifunctional Evaluation of Relative Mixture Effects

**FIGURE 2:**
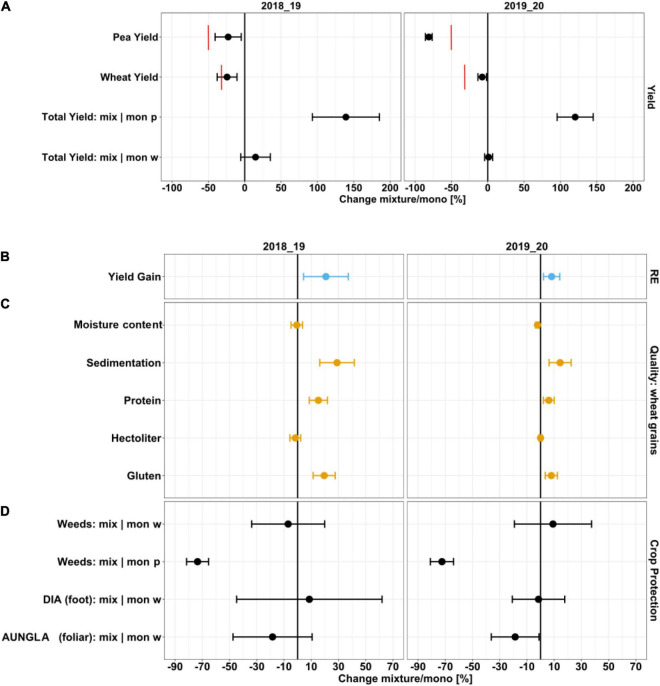
Mixture effects on **(A)** yield, **(B)** yield gain as a measure of resource efficiency (RE), **(C)** quality parameters of wheat, and **(D)** crop protection. Red bars indicate expected yield levels of wheat and pea-based on sowing densities in mixtures compared to pure stands. Estimated marginal means and 0.95 confidence intervals were derived from nlmes. Total yield and weeds are referred to either relative to wheat monocultures (mix|mon w) or relative to pea monocultures (mix|mon p).

Due to failure of the peas in the 2017/18 season, relative mixture effects could only be analyzed for 2018/19 and 2019/20 (for statistical summary, see Supplementary Tables 2, 3). Based on sowing frequencies, expected wheat yields were 70% and pea yields 50% of the respective pure stands. Yields of wheat compared to pure stands at full sowing density were 75.7% in 2018/19 and 92.4% in 2019/20, and for peas 77.2% in 2018/19 and 18.9% in 2019/20. The RMEs of total yields compared to wheat in the monocultures were close to 100% in both years while compared to peas in the monocultures they were 139 and 120% in 2018/19 and 2019/20, respectively (Figure 2A). Relative yield gain was 20.8% in 2018/19 and 8.1% in 2019/20 (Figure 2B and Supplementary Table 3). Sedimentation and protein and gluten content of wheat were considerably higher in the mixtures in both years and had greater effects in 2018/19 (Figure 2C and Supplementary Table 3). RMEs for weed cover were especially high compared to pea in the monocultures but agronomically irrelevant with respect to wheat as weed levels in wheat were very low (Figure 2D). RMEs for AUNGLA and DIA in wheat were moderate to low in both years except for a 19% reduction in AUNGLA in 2019/20.

### Wheat Entry and Interaction Effects

Lodging in the wheat monocultures and mixtures was also low in both seasons (max 3.8% in the “Liocharls” monoculture 2020). In the pea monocultures, however, lodging was significantly higher than in all the mixtures in both years (75 vs. 3% in 2018/19 and 29 vs. 1.6% in 2019/20).

Growing system (mixed vs. monoculture) and wheat entry indicated significant system × entry interaction effects for yield, total mixture yield, wheat protein content, AUNGLA, and weeds in at least one of two seasons (2018/19 or 2019/20). However, the main effects were usually more than one order of magnitude greater than the interaction effects. Relatively strong system × entry interactions occurred for total yield in 2018/19 with insignificant system effects. Similarly, the system × entry interaction was significant for weed cover in wheat in 2019/20 (Supplementary Table 5).

#### Crop Protection

Weed pressure in wheat was generally low in 2018/19 and 2019/20, with 1.8 and 2.3% weed cover in the mixtures and 1.9 and 2.5% in the pure stands. While the wheat entry × system interacted, at such low weed pressure this is biologically irrelevant. In the pea monocultures, weed cover was also relatively low in both years but significantly higher than in wheat and in comparison, to all mixtures with 7.3 and 9.2% (*P <* 0.01) in the two respective seasons (Data not shown).

**FIGURE 3:**
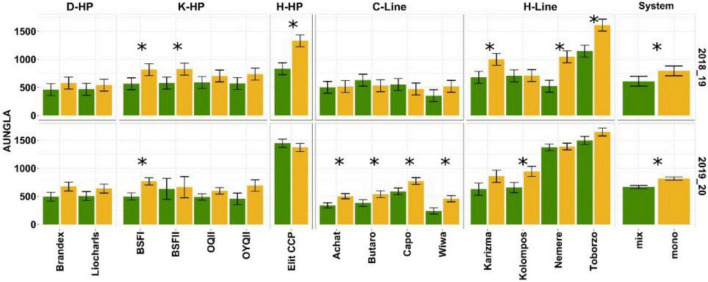
Area under the curve for non-green leaf area (AUNGLA). Wheat-pea mixtures are indicated in green and monoculture treatments in yellow. Estimated marginal means and standard error for the 2018/19 and 2019/20 seasons. Asterisks indicate significant differences at *P* < 0.05 between mixtures and monocultures in wheat entries estimated from lmes followed by pairwise comparison with Holm correction. Error bars indicate standard error.

In 2018/19, mean NGLA on the first assessment date was 4.2% in the monocultures and 3.6% in the mixtures. On the second assessment date, the mean NGLA was 17.7% in the monocultures and 13.2% in the mixtures. In 2019/20, mean NGLA on the first assessment date was 7.4% in the monocultures and 6.5% in the mixtures. On the second assessment date, mean NGLA was 37.3% in the monocultures and 29.6% in the mixtures. For both years and assessment dates, NGLA was mainly caused by senescence and *Drechsleratritici-repentis* (DTR), with respective incidences of nearly 100%. In very few cases, (2–3% in 2019 and 13% on the first assessment in 2020), stripe rust (*Puccinia striiformis*) was more prevalent than DTR. Mean AUNGLA for wheat in the mixtures was 23 and 19% lower than in the monocultures in 2019 and 2020, respectively (Figure 3). Wheat entry interacted significantly with system in 2018/19 [*F*_(14_, _87)_ = 4,996, *P* ≥ 0.01] but not in 2019/20. In both years, the main effects of entry and system were highly significant (*P* < 0.01) and in 2019 considerably larger than interaction (Supplementary Table 6), warranting a closer look at the main effects. However, there was no discernable pattern among or within entry groups within or across years (Figure 3).

Overall, cereal foot disease pressure was similar in both seasons, but no discernable mixture effect or lodging could be associated with it. Incidence of *O. yallundae* was highest at 0.77 and 0.82 in 2019 and 2020, respectively, while incidence of *Fusarium* spp. (0.27 and 0.11, respectively) and *C. cereale* (0.1 and 0, respectively) were rather low. Joint disease indices in 2018/19 were 47.6 in the monocultures and 47.3 in the mixtures, indicating moderate severity. In 2019/20, they were 46.7 for the monocultures and 44.8 for the mixtures. No mixture effects were detected.

#### Grain Yield and Quality

**FIGURE 4:**
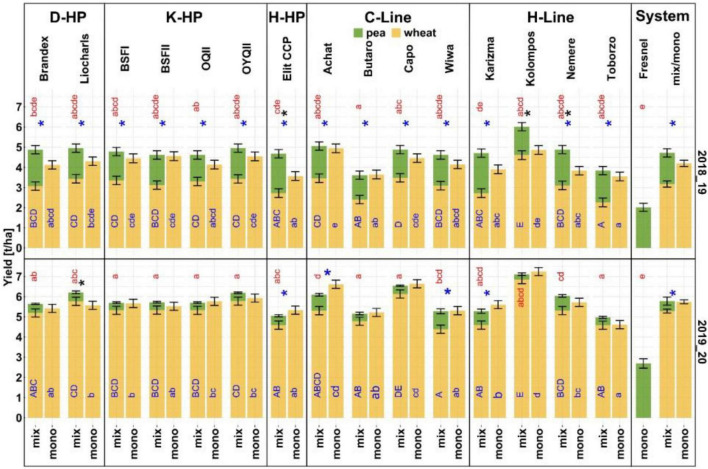
Yield in the mixtures and monocultures (2018/19 and 2019/20). Estimated marginal means and standard error from linear mixed effect models are plotted. Significant differences at *P* < 0.05 estimated from mixed models with pairwise comparison and Holm correction are shown. Small blue letters indicate significant differences among wheat entries in the monocultures and blue capital letters among the wheat cultivar mixtures. Red letters indicate significant differences in pea yield. Asterisks indicate significant differences in wheat yield (blue) and total yield (wheat + pea) (black) between systems.

Overall mean wheat yield in the monocultures and mixtures was 4.2 and 3.2 t ha^–1^ in 2018/19 and 5.8 and 5.3 t ha^–1^in 2019/20, respectively. In both seasons, the mean wheat yield in the pure stands was significantly higher than in the mixtures that had been sown at 70% seed density (Figure 4). The respective mean pea yield in the monoculture and mixtures was 2 and 1.5 t ha^–1^in 2018/19 and 2.7 and 0.5 t ha^–1^ in 2019/20. While significant system × entry interactions were found, the main effects were considerably greater than the interactions (Supplementary Table 7).

Despite the lower monoculture yield of peas in 2018/19, the contribution of peas to total mixture yield was considerable in that year (1.2–2 t ha^–1^), with no significant reductions in pea yield through mixing in 10 out of 15 cases. In response, almost all wheat cultivars except “Kolompos” indicated significantly lower yield in the mixtures than in the monocultures (Figure 4). Wheat yield in the monocultures ranged from 3.6 (“Toborzo”) to 4.9 t ha^–1^ (“Achat”) and in the mixtures from 2.3 (“Toborzo”) to 4.6 t ha^–1^ (“Kolompos,” Figure 4). Ranks in the monocultures for total yield were different to the ranks for wheat yield in the mixtures, with the lowest total yield of 3.6 t ha^–1^in the “Butaro” pea mix and highest in the “Kolompos” pea mix (6 t ha^–1^). Mixtures with “Elit CCP,” “Kolompos,” and “Nemere” had significantly higher total yield than the wheat monocultures (Figure 4).

In 2019/20, wheat yield in the monocultures ranged from 4.6 (“Toborzo”) to 7.3 t ha^–1^ (“Kolompos”) and in the mixtures from 4.4 (“Wiwa”) to 6.9 t ha^–1^ (“Kolompos”). While pea monoculture yield was higher than in 2018/19, the contribution of peas to total mixture yield was very low in 2019/20 (0.3–0.9 t ha^–1^), and peas yielded significantly less in all the mixtures than in the monocultures (Figure 4). “Achat,” “Elit CCP,” “Karizma,” and “Wiwa” yielded significantly lower in the mixtures than in the monocultures (Figure 4), resulting in rank changes between systems and, thus, interaction effects. Total yield (wheat + pea) was significantly affected by wheat entry [*F*_(14_, _84)_ = 22.4, *P* < 0.01] and ranged from 5 (“Toborzo,” pea) to 7.1 t ha^–1^ (“Kolompos,” pea). With one exception of “Liocharls” in the mixtures, total pea-wheat yield did not differ from wheat monoculture yield (Figure 4).

**FIGURE 5:**
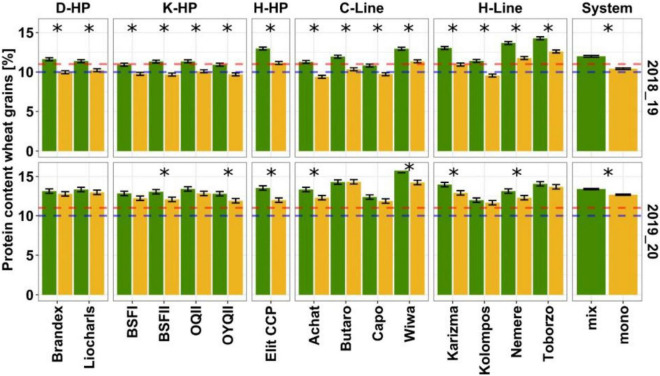
Wheat grain protein content (%) in the mixtures (green) and monoculture (yellow). Estimated marginal means and standard error for from linear mixed effect models are plotted. Asterisks show significant differences at *P* < 0.05 estimated from mixed models with pairwise comparison and Holm correction between the mixtures and monocultures in wheat entries. Dashed lines indicate quality thresholds for protein given by Drangmeister (2011). Red dashed line marks 10% and the blue dashed line 11% protein content. Quality classes are as follows: fodder wheat < 10%, baking wheat = 10–11%, and high-quality baking wheat > 11%.

In 2018/19, mean protein content in the mixtures was 12.3% (range: 10.8, “Capo”; 14.3%, “Toborzo”) and 10.8% in monocultures (range: 9.4, “Achat”; 12.6%, “Toborzo”). All the wheat entries significantly increased their protein content in the mixtures compared to monocultures, and apart from entries that had already reached the baking quality class in monoculture (“Wiwa,” “Karizma,” “Nemere,” and “Toborzo”), the remaining wheat entries increased their protein ranking class from fodder to baking wheat or from intermediate baking to top baking quality (Figure 5). Although a significant but weak interaction occurred between system and wheat entry [*F*_(14_, _84)_ = 2, *P* = 0.025], main effects of entry and growing system were much greater (Supplementary Table 8). In 2019/20, wheat grain protein content in the mixtures was 13.7% and in the monocultures 12.9%, ranging in the mixtures from 12 (“Kolompos”) to 15.8% (“Wiwa”) and in the monocultures from 11.7 (“Kolompos”) to 14.3% (“Butaro”). As all the entries were classified as top baking quality in the monocultures, no improvement in baking quality classification was found between the two growing systems.

**FIGURE 6:**
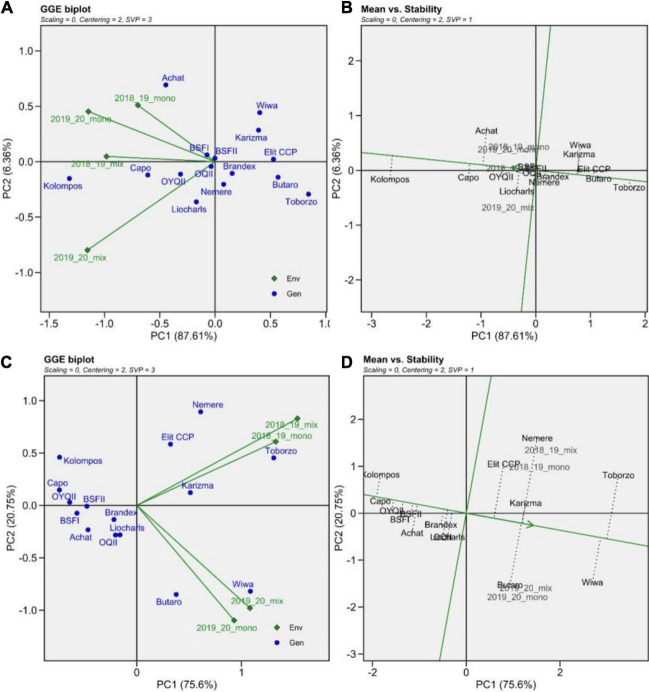
GGE biplots for wheat **(A)** yield and **(B)** stability and protein **(C)** content and **(D)** stability across seasons 2018/19 and 2019/20 seasons. For the symmetrical (SVP = 3) GGE biplot entries are indicated in blue and environments in green. For the mean vs. stability biplot, entries are black and environments are gray.

In the genotype main effect plus genotype-by-environment interaction analysis (GGE), the GGE explained 95% of wheat yield variation and 96% of variation in wheat grain protein (Figures 6A,B). First, the GGE was applied to assess entry association with cropping system and seasons defined as environments. For yield, the HPs (except for “Elit CCP”) clustered close to the biplot origin, indicating less yield variability across all environments (Figure 6A). “Kolompos” showed the strongest association with all environments, while Achathad a strong yield advantage in both monoculture environments and “Capo” and “OYQII” in the mixtures. The yield performance of “Wiwa,” “Elit CCP,” “Toborzo,” “Butaro,” and “Brandex” was not associated with any specific environment.

The mean vs. stability plots (Figures 6B,D) allowed for the assessment of genotype performance and stability ranking across environments. The green horizontal axis is called the average environment axis (AEA) and ranks the genotypes by their yield performance. The average environment coordination (AEC) axis is the second green axis that runs through the biplot origin and perpendicular to the AEA; the farther the distance from the origin of the biplot along this line, the greater the instability of the genotype, which means that the longer the length of the line or vector connecting the genotype to the AEA, the higher the GE interaction and the greater the instability of the genotype in all environments. Overall, it is conspicuous that almost all the HPs, with the exception of “Liocharls,” display greater yield stability than the majority of the line cultivars (Figure 6B).

In the protein-GGE, the experimental seasons were not associated with each other; however, both cropping systems in each experimental season were similar for protein. Additionally, different entries were strongly associated with the two experimental seasons for protein content (Figure 6C). The entry pattern for protein GGE is opposite to that of yield analysis. Here, “Butaro” and “Wiwa” are associated with both systems in 2019/20, while “Toborzo,” “Nemere,” “Elit CCP,” and “Karizma” are associated with both systems in 2018/19. With the exception of “Elit CCP,” the HPs displayed highest stability in protein content with some of the line cultivars indicating strong association to specific environments (Figures 6C,D).

#### Yield Gain and Land Equivalent Ratio

**FIGURE 7:**
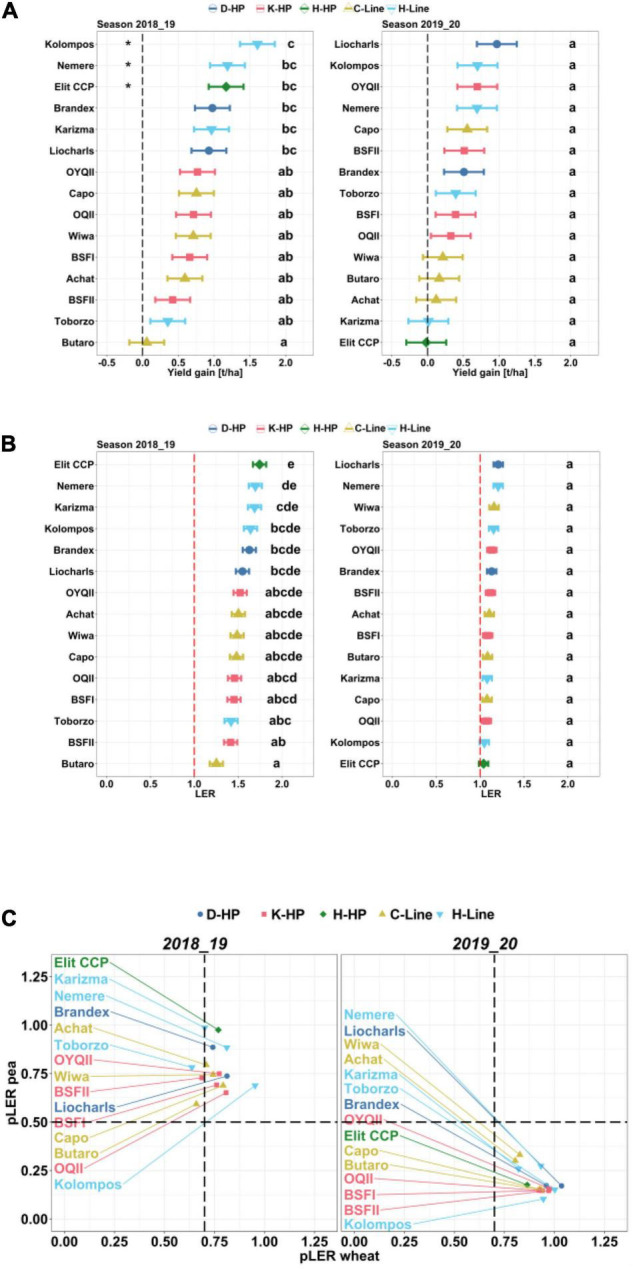
**(A)** Estimated marginal means and standard error for yield gains, **(B)** land equivalent ratios (LER), and **(C)** partial LER of wheat and peas of the 2018/19 and 2019/20 seasons. Significant differences at *P* < 0.05 estimated from mixed models with pairwise comparison and Holm correction are shown. Letters indicate significant differences between wheat entries. Stars indicate significant differences in yield gain from zero. The vertical dashed line in C represents the sowing ratio of wheat (mix/mono) and the horizontal line the sowing ratio of pea (mix/mono).

In 2018/19, yield gain (YG) was 0.8 t ha^–1^, equaling a relative yield increase of 19% compared to the monocultures, with significant wheat entry effects [*F*_(14_, _42)_ = 5.7, *P* = 0, Supplementary Table 9]. YG ranged from 0.1 (2.4%, “Butaro”) to 1.6 t ha^–1^ (38%, “Kolompos”; Figure 7A), and it differed significantly from zero for “Kolompos” (1.6 t ha^–1^), “Nemere” (1.2 t ha^–1^), and “Elit CCP” (1.2 t ha^–1^), all from Hungary.

In 2019/20, the mean YG across entries was only 0.4 t ha^–1^(7% yield increase) with no significant differences between entries or from zero (Figure 7A). Relative yield gain ranged from 0.3(“Elit CCP”) to 16.7% (“Liocharls”). In contrast to 2018/19, when the Hungarian entries dominated the top ranks, no specific group dominated in 2019/20. Conspicuously, the D-HP and K-HP-based mixtures resulted in similar YG in both years, which was not the case for H-HP or the Hungarian cultivar mixtures. For example, “Elit CCP” over-yielded in 2018/19 by 1.2 t ha^–1^ but not in 2019/20. Thus, the range of over-yielding in these two contrasting seasons was 1.2, i.e., twice as high as the mean YG (0.6 t ha^–1^). In contrast, mixtures with “OYQII” over-yielded by 0.75 and 0.7 t ha^–1^ in the 2 years; thus, over-yielding varied by only 0.05 t ha^–1^, albeit with different fractions of wheat and peas in the different years (Figure 4).

Although all the LER values were > 1 in both years, the effects were considerably greater in 2018/19 (Figure 7B), and entry effects were significant [*F*_(14,. 42)_ = 6.5, *P* = 0, Supplementary Table 9], with almost the same ranking as for the YG. In 2019/20, LER ranged from 1 (“Elit CCP”) to 1.2 (“Liocharls”) (mean 1.1), with no significant entry effects explaining the apparent dissimilarity in the ranks of YG and LER in that year. Most conspicuously, while YG in mixtures with “Kolompos” was second highest, the LER of these mixtures was second-lowest (Figure 7B). Nevertheless, LER and YG were highly correlated in both 2018/19 (*r* = 0.9, *P <* 0.01) and 2019/20 (*r* = 0.86, *P <* 0.01).

Effects of wheat on peas and peas on wheat were visualized by plotting the partial LERs per species against each other (Figure 7C). For peas at sowing density of 50%,pLER > 0.5 indicates over-yielding, while for wheat (sowing density 70%), pLER > 0.7 indicates over-yielding. In both seasons, the wheat entries significantly varied for their pLER and their effects on the pLER of peas (Supplementary Table 9). However, while wheat and pea pLERs in 2018/19 were greater than the expected pLER, although with few exceptions, in 2019/20, the pLERs of wheat were greater and those of peas were lower than the expected values (Figure 7C).

### Wheat Traits

In the set of evaluated entries, there was a considerable variation in days to heading (DTH, BBCH 50) due to the inclusion of Hungarian and central European entries. Mean DTH in the 2018/19 season was 224 and 208 days in 2019/20, corresponding to the fact that the experiment was sown 2 weeks earlier in 2018/19. Entry had by far the strongest effect on DTH in both years. A significant systems effect was found in 2019/20, while in 2018/19 the system × entry interaction was small. The system × entry interaction effects were one or two orders of magnitude smaller than the entry effects (Supplementary Table 11). The relative ranking of the entries was consistent across both years (Supplementary Table 12). The Hungarian entries were the earliest, and the organic line cultivars and “Achat” were the latest. The K-HP entries and “Capo” constituted a group of mid-early entries relative to the entire entry set. “Kolompos” was the latest Hungarian entry and grouped with the mid-early entries in 2018/19.

**FIGURE 8:**
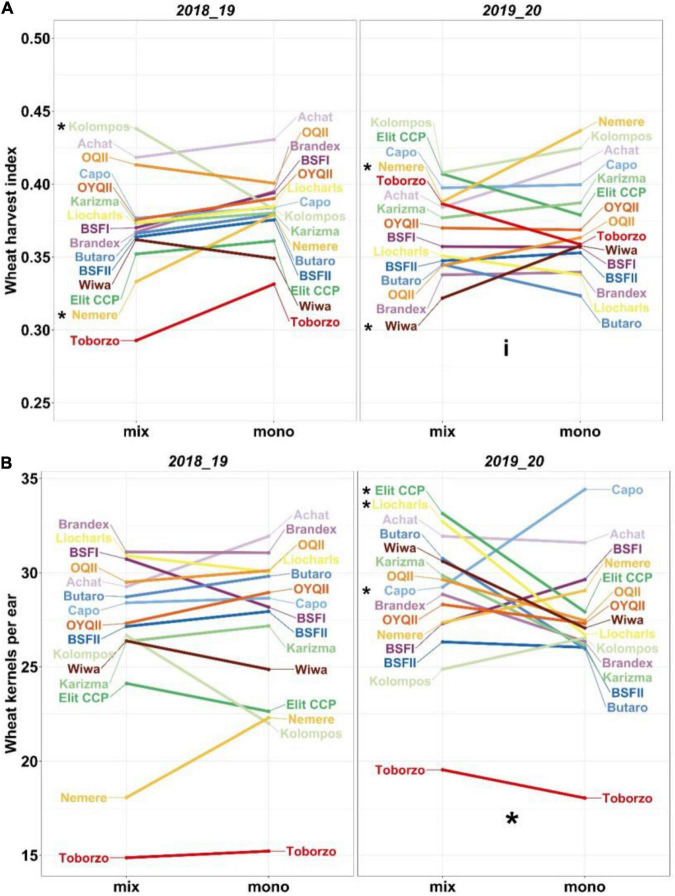
Interaction plots for **(A)** harvest index and **(B)** kernels per ear. Estimated marginal means from linear mixed effect models are plotted. Small stars indicate significant differences in entries between systems and large stars between systems across entries, and “i” indicates significant interactions at *P* < 0.05 estimated from mixed models with pairwise comparison and Holm correction.

Changes in HI were generally small and mostly insignificant. Reactions of the wheat entries were very variable and sometimes contrasting in the two seasons. In the monocultures, the ranking of the entries differed between years, e.g., “Nemere” and “Kolompos” were intermediate in 2018/19 but highest in 2019/20. In mixture with pea, the HI of “Nemere” was significantly lower in 2018/19 (mix: 0.33, mono: 0.38) and 2019/20 (mix: 0.39, mono: 0.44), while that of “Kolompos” was significantly higher in 2018/19 (mix: 0.44, mono: 0.38) but was reduced in 2019/20 (mix: 0.41, mono: 0.43). For “Wiwa” and “OQII,” HI showed a similar pattern to “Kolompos” but in contrast to “Elit CCP” (Figure 8A).

In mixture with pea, the different wheat entries indicated high variability for kernel number per ear depending on the experimental year. Entry responses indicated a similar pattern to HI with the exception of “Wiwa,” which, despite a significant reduction in HI in the mixtures in 2019/20, slightly increased its number of kernels per ear in that year. “Kolompos” indicated an increase in kernel number per ear in the mixtures in 2018/19 (Figure 8B and Supplementary Table 13).

**FIGURE 9:**
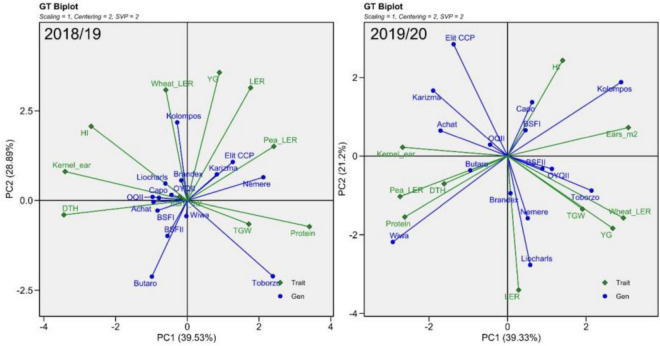
Genotype by trait biplots for two seasons. Entries are indicated in blue and traits in green.

The GT biplot (genotype by trait) explained 68% of the total trait variation in 2018/19 and 61% in 2019/20 (Figure 9). The interpretation is similar to that of the GGE biplot of yield and protein (Figure 6), in that the cosine of the angle between two traits approximates the correlation between them. Additionally, the length of the vectors indicates closeness of association with other traits. Traits with shorter vectors tend to have weaker associations with other traits. The same is true for genotypes and traits in terms of cosine of the angle between the genotypes and the traits. Genotypes found closely located (< 90°) to specific traits indicate high propensity to a trait or traits, referred to as genotype trait profile. If trait profiles between genotypes are different, they represent contrasting trait profiles.

Of interest is the question of how the measured traits (HI, kernels per ear, ears m^–2^, and DTH) interact and how they affect the yield and quality performance of the entries in mixtures (LER, YG, TGW, and protein). The number of kernels per ear and HI had vectors of similar length in both years, and the cosine angle indicates that they were correlated in 2018/19 but not in 2019/20. DTH was correlated with both kernels per ear and HI in 2018/19; however, ears m^–2^ was not associated with these traits. In 2019/20, DTH was still correlated with kernels per ear. In addition, HI and ears m^–2^ were positively correlated; however, these two traits were negatively correlated with the former two (Figure 9).

With respect to yield performance, it is not surprising that wheat pLER, YG, and LER are all closely associated. However, while pea pLER grouped with the aforementioned traits in 2018/19, it was weakly correlated with LER and negatively with YG in 2019/20, the year when more nitrogen was available in the soil. Nevertheless, with respect to quality, protein content was always closely associated with pea pLER. In contrast, HI was always negatively related with protein content, while it was correlated strongly with wheat pLER in 2019/20. This indicates that HI directly enhanced wheat competitiveness. The negative effect on pea pLER is the logical consequence. In contrast to HI, kernels per ear and days to heading were negatively associated with protein content in the first but positively associated in the second year. In 2019/20, both TGW and ears m^–2^ correlated with wheat pLER.

As depicted by the longer blue vectors, entry trait associations were stronger in 2019/20 than in 2018/19. Three Hungarian pure line cultivars and “Butaro” indicated stronger associations to plant traits in the first year. In 2019/20, most pure line cultivars, the H-HP “Elit CCP,” and the D-HP “Liocharls” indicated stronger associations to specific traits. The K-HPs, in general, tended toward the GT biplot origin in both years (Figure 9).

## Discussion

The mixture effects found in this study are confirmed by previous reports on cereal-legume mixtures with respect to improved cereal grain quality, weed suppression, resource use efficiency (Bedoussac et al., 2015), yield gain (Li et al., 2020), and lodging resistance (Kontturi et al., 2011; Podgórska-Lesiak and Sobkowicz, 2013). The two cropping seasons, 2018/19 and 2019/20, when the pea-wheat mixtures could be realized were relatively similar with respect to moderate to low water availability and temperature conditions but differed mostly in nitrogen availability. In 2018/19, when nitrogen was deficient, wheat baking quality could be improved to a high-quality baking standard. In 2019/20, when nitrogen provision was more adequate, standard high baking quality could be already achieved in the pure stands, while in the mixture’s protein content was increased even further. Weeds and foliar diseases were generally reduced, albeit only moderately, because of low weed and disease pressure and no specific resistance-related differences to foliar pathogens became evident in wheat under these conditions. The fact that senescence, overall, was delayed in the mixtures, reducing the area under the non-green leaf area curve (AUNGLA) points to beneficial interactions due to changes in wheat density and the addition of peas with respect to resource use dynamics over time. No effects on foot diseases were found, as it is often the case when studying mixtures (Finckh and Wolfe, 2015).

In reaction to the differing nitrogen supply, wheat entries displayed a broad range of reactions to growing in pure stands and wheat-pea mixtures with respect to yield and quality on the one hand. For example, in 2018/19, earliness could be one explanation for high YG in some Hungarian entries, except for “Toborzo” that seemed to be too early, while “Butaro,” the latest entry, had the lowest YG in this season. Earliness was strongly associated with kernels per ear and HI in 2018/19; however, in 2019/20, HI was affected negatively by earliness. Depending on the year, different traits, such as harvest index (HI), days to heading (DTH), and ears m^–2,^ were correlated with the performance traits of wheat and peas, such as LER or partial LER, yield gain (YG), and wheat grain protein content. Nevertheless, in both experimental years, the HI of wheat appeared to be a robust measure for predicting effects on pea performance (pea pLER) and, subsequently, on wheat quality in mixtures, as protein content was negatively correlated with HI and positively with pea pLER.

With the exception of the Hungarian “Elit CCP,” independent of the growing season, the HPs displayed highest stability with respect to yield and quality and no specific trait profiles. This indicates that while selection of specific traits might be of use when selecting pure line cultivars for mixtures, this avenue might not be the best when dealing with HPs. The high stability of the HPs was at an overall intermediate level for yield and protein values. Some of the Hungarian line cultivars achieved either high yield or protein content in the two experimental years. This was due to the fact that weather conditions, overall, were very warm and similar to more continental summers. Increased environmental variability and weather extremes challenge future agricultural production and need to be mitigated. Therefore, HPs can add an additional level of environmental stress-buffering capacity to wheat-pea species mixtures.

The multicriteria evaluation confirms the relevance of inter- and intra-specific diversity for multifunctionality as was previously reported for multifunctional grasslands (Isbell et al., 2017, 2011). Such a multifunctional perspective in agriculture is by no means established in practice. Rather, the focus, even in organic agriculture, is on yield of single crop species. This is, in large part, due to lack of established value chains based on mixed cropping (Kiær et al., 2022, this volume).

### Entry Effects and System × Entry Interactions of Performance

Although system × entry interactions were mostly weak compared to the main effects of the wheat entries, they were generally greater in 2018/19 than in 2019/20. Water availability was likely somewhat lower in 2018/19 than in 2019/20 because of the extreme drought in 2017/18. Complementarity, with respect to water use between wheat and pea, likely played a role. Thus, differences in YG and LER among the wheat entries were only significant in 2018/19.

The complementarity of legumes and cereals with respect to nitrogen pools accessible by cereals (soil) and legumes (soil and atmospheric nitrogen), and different competitive abilities with respect to soil nitrogen of cereals (high) and legumes (low) depend on these resources being limited (Bedoussac et al., 2015), which was most likely the case in the 2018/19 season. Under the relatively low nutrient levels in 2018/19, peas were more competitive and contributed with 33% more than expected to total yields in mixture compared to monoculture. In contrast, in 2019/20, total yield was 20% higher than in the previous year, but peas were suppressed by wheat, and their contribution to total yield was only 9%, with strong effects of the wheat entries on pea performance. The fact that growing system and entry had similarly strong effects in 2018/19, but entry effects were stronger than system effects in 2019/20 seem to confirm other studies (Moutier et al., 2021) that competitive interactions among peas and wheat were strongly driven by nitrogen availability. The spatial and temporal dimensions of niche differentiation, such as different rooting patterns and plant phenology, are, here, of particular relevance (see next section). It should be noted that even the higher N-levels in the second year were relatively low compared to conventional farming systems. The K-HPs that were bred for and selected in organic systems often outperform line cultivars under organic but not under conventional conditions (Weedon and Finckh, 2019, 2021). Nevertheless, wheat yield is more predictable under high nutrient conditions. LER seems more sensitive to cultivar variation; however, the overall picture is similar to YG. Leveling of cultivar-based performance differences also makes it challenging to make robust statements with respect to different entry groups and relationships between traits and performance.

The GGE analysis revealed that the K-HPs and “Brandex” had a lower variation for grain yield and protein than “Elit CCP,” C-lines, and H-Lines. With respect to yield performance in mixtures with the pea cultivar “Fresnel” and the monocultures “Kolompos,” “Achat,” and “Capo” (the former two are conventional relatively short cultivars compared to the other entries evaluated) performed best in the monocultures. In the mixtures, they also performed well; however, “Achat” did so less. The other line cultivars varied greatly among years in their relative performance. In contrast, with the exception of the Hungarian “Elit CCP,” in 2018/19, the HPs were only outperformed in the pure stands by “Achat” and in the mixtures by “Kolompos.” In 2019/20, the HPs were mostly outperformed by “Achat,” “Capo,” and “Kolompos” in the pure stands while in the mixtures only “Kolompos” out-yielded all the HPs. Thus, while most of the HPs, with the exception of “Elit CCP” were well suited for mixtures, among the pure line cultivars, “Kolompos” and “Capo” appeared particularly well suited with respect to yield.

Using the 4C approach (Justes et al., 2021), the pLERs allowed us to draw conclusions with respect to competition, complementarity, compensation, and cooperation. In 2018/19, all the wheat entries except for “Toborzo,” “BSFII” and “Butaro” fall in the top right section of the pLER plot, indicating that complementarity and cooperation were stronger than the competition. In 2019/20, competition was stronger than cooperation and complementarity, and wheat suppressed the peas. Still, in 2018/19, pea pLER was highest in combination with four of the five best-performing entries (“Karizma,” “Elit CCP,” “Nemere” and “Brandex”). Pea pLER was somewhat reduced with “Kolompos,” but the latter had the highest wheat pLER. All the other wheat entries indicated greater reduction in yield in relation to pea. This points to overall asymmetric competitive interactions depending on wheat entry (Weiner, 1990). In parallel to the pLERs, HI varied considerably. In 2018/19, most of the entries did not differ significantly in HI between systems, indicating similar competition in the mixtures and monocultures, while in 2019/20 some interactions occurred. In contrast, the HI of “Kolompos” increased in the mixtures compared to the monocultures in 2018/19 but not in 2019/20, while the HI of “Nemere” decreased in the mixtures in both years. All the other entries had no significant changes in their HI. This confirms a differential competitive response of wheat entries with respect to resource allocation and depending on environment, indicating different routes to high LER or *YG* values that ideally should be exploited.

In contrast to yield, the most important factor affecting protein content of wheat within the year was growing system (i.e., mixed cropping), but the increase in protein content in mixture with pea was almost twice as high under low nitrogen levels (+1.5%) compared to higher nitrogen levels (+0.7%), and significant system × entry interactions only occurred in 2018/19. The line cultivars “Wiwa” and “Toborzo” outperformed the HPs in terms of protein content but not yield across environments. The reversed pattern of yield and protein GGEs confirm the well-known trade-off between yield and quality in wheat (Michel et al., 2019) and the fact that the effect of cereal-legume mixtures on wheat quality is almost entirely due to nitrogen (Bedoussac et al., 2015). Nevertheless, the increase in protein content in both years, particularly 2019/20, was greater in the pure line cultivars than in the HP entries, except for “Elit CCP.” The fact that HP entries are heterogeneous results in higher variation in protein potential. The German HPs bred for good baking quality (“Brandex,” “Liocharls,” and: “OQII”) had higher protein content in the pure stands under high N-input in the second year than the more diverse HPs, “OYQII,” and the two BSF’-HPs. However, in the mixtures, these differences were no longer evident, suggesting greater plasticity of the HPs for that trait. Thus, it appears that under high nitrogen input levels, the relative yield performance of wheat when mixed with a determinate pea cultivar is quite predictable from the wheat pure stands. In contrast, selection for improved mixture performance and improved protein levels of wheat in the mixtures with peas may only be useful under low nitrogen levels to identify entries highly efficient for nitrogen use.

While foliar disease levels were very low in our experiments, leaf senescence played a more prominent role and was the main factor contributing to the non-green leaf area. AUNGLA, as an indicator for leaf senescence, was reduced in the mixtures compared to the monocultures in 2018/19 and 2019/20. Delayed senescence could be a possible explanation for higher protein contents, since the onset of senescence explained up to 86% of the variation in nitrogen utilization efficiency in wheat (Gaju et al., 2011).

In contrast to yield and protein effects, with respect to weeds, system × entry interactions were high under higher nitrogen levels, even if absolute weed levels were low. Weed pressure is increased by high nutrient levels and is an important issue not only in organic growing systems, but particularly in species mixtures, as herbicides are usually incompatible with such mixtures. Thus, improved weed suppression and management through optimization of weed-suppressive crop mixtures and associated breeding programs should be highlighted as important aims to improve crop mixtures and their wider application.

### Wheat Trait Effects on Mixture Performance

Increased competition through increased plant density or reduced resources in monocultures increases the allocation of resources to vegetative relative to reproductive plant organs, leading to lower HI in wild plants (Keddy, 2017, p. 128) and arable crops (Li et al., 2015). Chen et al. (2020) found a greater increase in total biomass than in grain yield in mixtures, because in mixtures reproductive effort is reduced (lower harvest index) relative to monocultures because of increased competition. Nevertheless, this does not necessarily indicate a trade-off, as is demonstrated with diversified wheat populations (Weedon and Finckh, 2021), and HI may, therefore, be a valuable indicator of reduced competition and increased complementarity in mixtures. Exploiting reduced competition and increased allocation to grains in mixtures may be a route for breeding to increase mixture performance in terms of grain yield (Chen et al., 2020).

The significantly increased harvest index of Kolompos in 2018/19 coincided with increased kernels per ear in the mixtures, and the reverse was true for Nemere. Even though these changes in kernels per ear were not statistically significant, the coherent contrasting pattern of both entries for the two variables suggests they might explain the changes in HI. It is possible that the reduced investment in the competition of Kolompos in the mixtures resulted in more kernels per ear, increasing its HI, while the presence of pea apparently played no significant role. Likely, the phenology of the mid-early Kolompos, relative to the set of evaluated entries, is complementary to the pea cultivar “Fresnel” contributing to (temporal) niche differentiation and, thus, reduced competition. Interestingly, “Kolompos” also had the longest seminal roots in a recent hydroponic study, even longer than those of wheat plants selected specifically for long roots (Timaeus et al., 2021). As noted before, in our experiment, mixture effects are due to species mixing and density effects in an absolute sense, but comparing different entries in the same experimental setup still enables some insights. Temporal niche differentiation, as indicated by differences in maximum daily growth rates of component species, is an important driver of yield gain in mixtures of canola with soybean or maize (Dong et al., 2018) and mixtures of maize with small-grain cereals or legumes (Li et al., 2020). These studies compared different crop species combinations. Only few studies have at least, in part, attempted to investigate the effect of intraspecific variation of crop species phenology on mixture performance as pointed out by (Demie et al., 2022, this volume). To breed new line cultivars or HPs, we should systematically exploit the positive effects of temporal niche differentiation.

Experimental studies show that early vigor has a positive impact on nitrogen use efficiency (Liao et al., 2004) and, therefore, likely on protein content. As pointed out above, earliness was not always beneficial among the wheat entries. In contrast to Moutier et al. (2021) who found that wheat cultivar earliness is significantly correlated with wheat protein content increase, we did not detect a significant correlation between DTH and protein. Thus, in 2018/19, the very early cultivar “Toborzo” had, by far, the highest protein content, followed by “Nemere,” “Karizma,” and “Elit CCP.” The mid-early cultivar “Kolompos” had rather low protein content values, likely due to its high yield, while “Wiwa,” a late cultivar, had comparatively high protein values. The interactions between a range of different factors, such as DTH, speed of leaf senescence, yield level, and their effect on protein content, are not well explored.

### Future Avenues for Breeding Research

The magnitude of stresses on cropping systems will have strong impacts on the relevance of cropping systems diversified at multiple levels. Increased frequencies of extreme weather events (IPCC, 2021) and additional socioeconomic factors, such as increasing market price and regulation for synthetic nitrogen fertilizers, could lift these systems to high relevance for farming practice. This would justify increased efforts in breeding programs and breeding research for diversified cropping systems.

Both wheat line cultivars bred for monocultures and HPs that evolved in single-species populations can be used to harness the advantages of cereal-legume mixtures, as indicated by the multifunctional evaluation. HPs add additional performance stability to species mixtures under environmental stress but their genetic background and selection environment used for their evolution need to be taken into account (Weedon and Finckh, 2019, 2021). Interaction of diversity at the intra- and interspecific levels in cropping system performance needs further study, particularly in different environments. Research on species mixtures in multiple environments might profit from a stronger link to crop community ecology. Gliessman introduced the concept of crop communities for cropping systems and community ecology as a discipline to refocus agricultural management and research to harness emergent effects and properties of plant communities that can be used in agriculture (Gliessman, 1987, p. 161). Systematic empirical research on species mixtures combined with plant ecology might contribute to the development of crop community ecology as a basis for multifunctional cropping systems, as described by Litrico and Violle (2015).

Upscaling experimental research across environments and integration of new conceptual perspectives from ecology needs to be complemented by experiments that investigate diversification mechanisms and interactions related to plant traits in more detail, particularly those related to the 4C approach (Justes et al., 2021). Traits should not only be studied as targets for breeding but also as indicators of competition/complementarity and adaptation to species mixtures. For example, genotypes can systematically be screened for maintenance or even increased HI in mixtures indicating reduced susceptibility to competition. Some key results, such as differences in variation in the harvest index of “Kolompos” and “Nemere,” were detected by combining the results of *post-hoc* tests and visual interaction plots. Elucidating such variation in system comparisons can help to pinpoint mechanistic relationships between reduced competition between peas and specific wheat entries (increased HI in mixture), plant traits (phenology), and performance advantages under certain environmental conditions. Such experiments can support future breeding efforts to more systematically study traits and how they are influenced by the 4Cs to further reduce competition and increase complementarity in mixtures. Hitherto, intraspecific variation of phenology as a source of variation of niche differentiation between crop partners seems to be largely unexplored. Exploring variation in temporal niche differentiation could also increase resource efficiency and yield gains in high-input farming systems (Li et al., 2020).

Despite the many well-known advantages of mixed cropping, many obstacles for species mixtures currently preclude their adoption in general (Kiær et al., 2022). With respect to wheat-pea mixtures for food products, market and processing opportunities are rare to nonexistent. Innovations in cropping system design may help to address practical challenges. In relay mixtures, for example, sowing of crop species and their harvest is done in a staggered fashion, avoiding the issue of grain separation prevalent in other mixture designs. This is especially relevant, since some specialized food processors require the highest grain purity because of consumer allergies. This highlights the importance of specific mixed cropping system designs and the need for their systematic investigation and development while integrating plant ecology and agronomy (Brooker et al., 2021).

Harnessing the advantages of species mixtures in farming practice needs to go beyond the cropping system perspective. The multifunctional agronomic advantages identified here can only be harnessed in practice for food crops if major obstacles for food grain mixtures are addressed downstream of the food supply chain (Meynard et al., 2013, 2018). This includes the need for public support to establish sufficient grain sorting facilities, storage, and logistics in an emerging sector that needs to scale up. Lack of infrastructure may slow down or even impede the adoption of such farming practices (Mamine and Farès, 2020). Failing to account for these challenges in the food system will result in failure to harness mixture advantages as emphasized in the food-system turn in agroecology (Francis et al., 2003; Gliessman, 2015). For this reason, tailored research strategies that integrate plant ecology, agronomy, breeding science, and practical value chain aspects are urgently needed.

## Data Availability Statement

The raw data supporting the conclusions of this article will be made available by the authors, without undue reservation.

## Author Contributions

MF, OW, and JT conceptualized the study and contributed to methodology, validation, and writing, reviewing, and editing. JT and OW contributed to formal analysis. MF contributed to resources, project administration, and funding acquisition. JT contributed to data curation and visualization. JT and MF wrote the original draft. MF and OW contributed to supervision. All authors have read and agreed to the published version of the manuscript.

## Conflict of Interest

The authors declare that the research was conducted in the absence of any commercial or financial relationships that could be construed as a potential conflict of interest.

## Publisher’s Note

All claims expressed in this article are solely those of the authors and do not necessarily represent those of their affiliated organizations, or those of the publisher, the editors and the reviewers. Any product that may be evaluated in this article, or claim that may be made by its manufacturer, is not guaranteed or endorsed by the publisher.
