# Supplementary material for: Harnessing the Potential of Wheat-Pea Species Mixtures: Evaluation of Multifunctional Performance and Wheat Diversity
Source: Front Plant Sci. 2022 Mar 25;13:846237. doi: 10.3389/fpls.2022.846237 (PMC8990764; doi:10.3389/fpls.2022.846237)
Supplement: Supplementary file 1 [file Data_Sheet_1.docx]

Supplementary Material

# Collected data and study design

Table S 1 Data collected during two experimental seasons. NGLA: non-green leaf area, WAS: weeks after sowing.

| **Data** | **Season 2018/19** | | **Season 2019/20** | |
| --- | --- | --- | --- | --- |
|  | **Date** | **Cereal BBCH** | **Date** | **Cereal BBCH** |
| Sowing | 16.10.2018 | 0 | 31.10.2019 | 0 |
| Emergence [plants / m^2^] | 23.11.2018 | 9 | 05.12.2020 | 9 |
| Weed cover [%] | 27.03.2019 | 23 | 27.03.2019 | 20-25 |
| Foliar disease [AUNGLAC , %] | 04.06., 17.6.2019 | 50-61, 71-77 | 16.06., 13.07.2020 | 71-73, 73-85 |
| Foot disease [DI] | 03.07.2019 | 70-81 | 7.7.2020 | 77-83 |
| Wheat biomass cuts | 01.08.2019 | 85 | 22.07.2020 | 85-87 |
| Lodging [%] | 02.08.2019 | 80-85 | 17.06.2020 | 71-73 |
| Grain harvest pure peas [t ha^-1^] | 23.07.2020 | 89 | 23.07.2020 | 89 |
| Grain harvest mixtures/pure wheat [t ha^-1^] | 06.08.2020 | 92 | 06.08.2020 | 92 |
| Protein content by NIRS [%] | 1.-10.9.2019 | 92 | 20.-24.8.2020 | 92 |
| Heading | 15.5.-5.6.2019 | 51-59 | 11.5.-6.6.2020 | 51-59 |

# Relative Mixture effects

Table S 2 Anova table from non-linear mixed effect models (nlmes) to estimate relative mixture effects. Numerator degrees of freedom (DF), response variable refers to the relative changes in mixture performances.

| **Year** | **Model Element** | **DF** | **F-value** | **P-value** |
| --- | --- | --- | --- | --- |
| 2018_19 | intercept | 1 | 13.8 | 0.000 |
| 2018_19 | response variable | 13 | 764.5 | 0.000 |
| 2019_20 | intercept | 1 | 94.7 | 0.000 |
| 2019_20 | response variable | 13 | 591.3 | 0.000 |

Table S 3 Relative mixture effects. Emmeans: Estimated marginal means; DF: degrees of freedom; CL: lower and upper 0.95 confidence limits. Derived from nlmes with pairwise comparison and Holm correction.

| **Year** | **Variable** | **Variable Type** | **Emmeans** | **DF** | **lower.CL** | **upper.CL** |
| --- | --- | --- | --- | --- | --- | --- |
| 2018_19 | AUNGLAC (foliar) | Crop Protection | -18.4 | 3 | -47.5 | 10.6 |
| 2018_19 | DIA (foot) | Crop Protection | 8.6 | 3 | -44.8 | 62.0 |
| 2018_19 | Gluten | Quality: wheat grains | 19.5 | 3 | 11.4 | 27.6 |
| 2018_19 | Hectoliter | Quality: wheat grains | -1.7 | 3 | -5.7 | 2.3 |
| 2018_19 | Pea Yield | Yield | -22.8 | 3 | -40.8 | -4.8 |
| 2018_19 | Protein | Quality: wheat grains | 15.2 | 3 | 8.6 | 21.9 |
| 2018_19 | Sedimentation | Quality: wheat grains | 28.9 | 3 | 16.3 | 41.6 |
| 2018_19 | Total Yield: mix\|mono pea | Yield | 139.2 | 3 | 93.0 | 185.3 |
| 2018_19 | Total Yield: mix\|mono wheat | Yield | 14.9 | 3 | -5.5 | 35.2 |
| 2018_19 | Water | Quality: wheat grains | -0.6 | 3 | -4.8 | 3.6 |
| 2018_19 | Weeds: mix\|mono pea | Crop Protection | -73.5 | 3 | -81.5 | -65.4 |
| 2018_19 | Weeds: mix\|mono wheat | Crop Protection | -7.0 | 3 | -33.8 | 19.8 |
| 2018_19 | Wheat Yield | Yield | -24.3 | 3 | -37.9 | -10.6 |
| 2018_19 | Yield Gain | RE | 20.8 | 3 | 4.4 | 37.2 |
| 2019_20 | AUNGLAC (foliar) | Crop Protection | -18.6 | 7 | -36.2 | -1.1 |
| 2019_20 | DIA (foot) | Crop Protection | -1.5 | 7 | -20.8 | 17.8 |
| 2019_20 | Gluten | Quality: wheat grains | 7.9 | 7 | 3.4 | 12.4 |
| 2019_20 | Hectoliter | Quality: wheat grains | 0.0 | 7 | -1.1 | 1.1 |
| 2019_20 | Pea Yield | Yield | -81.1 | 7 | -85.7 | -76.4 |
| 2019_20 | Protein | Quality: wheat grains | 6.1 | 7 | 2.1 | 10.0 |
| 2019_20 | Sedimentation | Quality: wheat grains | 14.4 | 7 | 6.3 | 22.5 |
| 2019_20 | Total Yield: mix\|mono pea | Yield | 120.3 | 7 | 95.5 | 145.1 |
| 2019_20 | Total Yield: mix\|mono wheat | Yield | 1.2 | 7 | -4.3 | 6.7 |
| 2019_20 | Water | Quality: wheat grains | -2.2 | 7 | -3.5 | -0.9 |
| 2019_20 | Weeds: mix\|mono pea | Crop Protection | -72.4 | 7 | -81.0 | -63.9 |
| 2019_20 | Weeds: mix\|mono wheat | Crop Protection | 9.1 | 7 | -19.2 | 37.5 |
| 2019_20 | Wheat Yield | Yield | -7.6 | 7 | -13.5 | -1.7 |
| 2019_20 | Yield Gain | RE | 8.1 | 7 | 2.2 | 14.1 |

# Absolute performance statistics

Table S 4 Anova for two-year models (2018_19/2019_20) for LER, yield gain and wheat and pea pLER. MS: Mean square error; DF: Numerator degrees of freedom; F: F – value; P: P- value from the linear mixed effects models for different agronomic traits of cereal entries. Entry represents the cereal entries.

|  | MS | DF | F | P |
| --- | --- | --- | --- | --- |
| **Yield Gain [t ha^-1^]** | | |  |  |
| Year (Y) | 0.6 | 1 | 3.3 | **0.00** |
| Entry (E) | 4.2 | 14 | 24.8 | **0.00** |
| Y x E | 0.3 | 14 | 1.9 | **0.04** |
| **LER** | |  |  |  |
| Year (Y) | 0.04 | 1 | 3.6 | **0.00** |
| Entry (E) | 5.19 | 14 | 474.5 | **0.00** |
| Y x E | 0.04 | 14 | 3.6 | **0.00** |
| **Pea pLER** | |  |  |  |
| Year (Y) | 0.05 | 1 | 6.5 | **0.00** |
| Entry (E) | 10.19 | 14 | 1356.9 | **0.00** |
| Y x E | 0.02 | 14 | 2.9 | **0.00** |
| **Wheat pLER** | |  |  |  |
| Year (Y) | 0.024 | 1 | 2.4 | **0.01** |
| Entry (E) | 0.835 | 14 | 85.0 | **0.00** |
| Y x E | 0.018 | 14 | 1.9 | **0.04** |

Table S 5 Ratios of mean squares of significant interaction effects (int) relative to the main effects system (S) or entry effects (E). Ns refers to non-significant interactions.

|  | **Lodging** | | **Weeds** | | **AUNGLA** | | **DIA** | |
| --- | --- | --- | --- | --- | --- | --- | --- | --- |
|  | **int/S** | **int/E** | **int/S** | **int/E** | **int/S** | **int/E** | **int/S** | **int/E** |
| 2018/19 | ns | | ns | | 0.08 | 0.16 | ns | |
| 2019/20 | ns | | 4.00 | 0.57 | ns | | ns | |
|  | **Wheat yield** | | **Total yield** | | **Protein** | |  |  |
|  | **int/S** | **int/E** | **int/S** | **int/E** | **int/S** | **int/E** |  |  |
| 2018/19 | 0.10 | 0.11 | 1.50 | 0.19 | 0.02 | 0.02 |  |  |
| 2019/20 | 0.17 | 0.09 | ns | | ns | |  |  |

Table S 6 Anova tables 2019/20 for foliar disease and weeds. MS: Mean square error; DF: Numerator degrees of freedom; F: F – value; P: P- value from the linear mixed effects models for different agronomic traits of cereal entries. Entry represents the cereal entries, System the cropping system (monoculture and mixture).

| **Season** | **Parameter** | **Factor** | **MS** | **DF** | **F** | **P** |
| --- | --- | --- | --- | --- | --- | --- |
| 2018/19 | AUNGLA | System (S) | 1031644 | 1 | 65396 | **0,00** |
|  |  | Entry (E) | 493947 | 14 | 31311 | **0,00** |
|  |  | SxE | 78813 | 14 | 4996 | **0,00** |
|  | Weeds in wheat [%] | System (S) | 0,64 | 1 | 4,5 | 0,13 |
|  |  | Entry (E) | 0,37 | 14 | 2,6 | **0,00** |
|  |  | SxE | 0,11 | 14 | 0,8 | 0,70 |
|  | Weeds in pea [%] | System (S) |  |  |  |  |
|  |  | Entry (E) | 8,0552 | 15 | 53,826 | **0,00** |
|  |  | SxE |  |  |  |  |
| 2019/20 | AUNGLA | System (S) | 1803202 | 1 | 43,5 | **0,00** |
|  |  | Entry (E) | 1575836 | 14 | 79,7 | **0,00** |
|  |  | SxE | 29638 | 14 | 0,9 | 0,46 |
|  | Weeds in wheat [%] | System (S) | 0,4 | 1 | 0,5 | 0,50 |
|  |  | Entry (E) | 2,8 | 14 | 3,1 | **0,00** |
|  |  | SxE | 1,6 | 14 | 1,8 | **0,05** |
|  | Weeds in pea [%] | System (S) |  |  |  |  |
|  |  | Entry (E) | 13,3 | 15 | 16,7 | **0,00** |
|  |  | SxE |  |  |  |  |

Table S 7 Anova tables for grain yields for two seasons. MS: Mean square error; DF: Numerator degrees of freedom; F: F – value; P: P- value from the linear mixed effects models for different agronomic traits of cereal entries. Entry represents the cereal entries, system the cropping system (monoculture and mixture).

|  | **2018/19** | | | | **2019/20** | | | |
| --- | --- | --- | --- | --- | --- | --- | --- | --- |
| **Wheat yield [t ha^-1^]** | | | | | | | | |
|  | MS | DF | F | P | MS | DF | F | P |
| System (S) | 2.1 | 1 | 22.4 | **0.01** | 1.8 | 1 | 12.5 | **0.04** |
| Entry (E) | 1.8 | 14 | 19.5 | **<0.01** | 3.2 | 14 | 22.5 | **<0.01** |
| SxE | 0.2 | 14 | 2 | **0.03** | 0.3 | 14 | 2.2 | **0.01** |
| **Pea yield [t ha^-1^]** | | | | | | | | |
| Entry | 0.2 | 15 | 5 | **<0.01** | 0.2 | 15 | 13.2 | **<0.01** |
| **Total yield [t ha^-1^]** | | | | | | | | |
| System (S) | 0.2 | 1 | 3.2 | 0.17 | 0 | 1 | 0.1 | 0.76 |
| Entry (E) | 1.6 | 14 | 22.9 | **<0.01** | 3 | 14 | 22.4 | **<0.01** |
| SxE | 0.3 | 14 | 3.9 | **<0.01** | 0.2 | 14 | 1.4 | 0.19 |

Table S 8 Anova tables for wheat grain proteins for two seasons. MS: Mean square error; DF: Numerator degrees of freedom; F: F – value; P: P- value. Derived from the linear mixed effects models for different agronomic traits of cereal entries. Entry represents the cereal entries, system the cropping system (monoculture and mixture).

|  | **2018/19** | | | | **2019/20** | | | |
| --- | --- | --- | --- | --- | --- | --- | --- | --- |
|  | **Protein wheat grains [%]** |  |  |  | **Protein wheat grains [%]** |  |  |  |
|  | MS | DF | F value | Pr(>F) | MS | DF | F value | Pr(>F) |
| System (S) | 11.1 | 1 | 109 | **0.00** | 15.8 | 1 | 50.3 | **0.00** |
| Entry (E) | 8.2 | 14 | 79.9 | **0.00** | 5.5 | 14 | 17.7 | **0.00** |
| SxE | 0.2 | 14 | 2 | **0.03** | 0.4 | 14 | 1.3 | 0.24 |

Table S 9 Anova for wheat entry effects on LER, pLER and yield gain (YG) for two seasons. MS: Mean square error, DF: numerator degrees of freedom, F: F – value, P: P – value, estimated from the linear mixed effects models.

|  | **2018/19** | | | | **2019/20** | | | |
| --- | --- | --- | --- | --- | --- | --- | --- | --- |
|  | MS | DF | F | P | MS | DF | F | P |
| **LER** | 0.07 | 14 | 6.5 | **0.00** | 0.01 | 14 | 1.4 | 0.22 |
| **Wheat pLER** | 0.02 | 14 | 3.4 | **0.00** | 0.02 | 14 | 6.5 | **0.00** |
| **Pea pLER** | 0.05 | 14 | 4.3 | **0.00** | 0.02 | 14 | 10.8 | **0.00** |
| **Yield Gain [t ha^-1^]** | 0.57 | 14 | 5.7 | **0.00** | 0.32 | 14 | 2.0 | **0.04** |

Table S 10 Comparison of cropping systems. Estimated marginal means (Emmeans), standard error (SE), degrees of freedom (DF) and lower and upper limits for the 0.95 confidence interval derived from lme/nlmes with pairwise comparison and Holm correction. Group refers to statistically significant differences at P < 0.05.

| **Year** | **Variable type** | **variable** | **System** | **Emmean** | **SE** | **DF** | **lower.CL** | **upper.CL** | **.group** |
| --- | --- | --- | --- | --- | --- | --- | --- | --- | --- |
| 2018_19 | Crop protection | AUNGLAC | mix | 611.00 | 87.2 | 3.1 | 256.00 | 966.00 | b |
| 2018_19 | Crop protection | AUNGLAC | mono | 796.00 | 87.2 | 3.1 | 442.00 | 1151.00 | a |
| 2019_20 | Crop protection | AUNGLAC | mix | 666.15 | 28.6 | 3.0 | 546.87 | 785.43 | a |
| 2019_20 | Crop protection | AUNGLAC | mono | 819.21 | 28.6 | 3.0 | 699.93 | 938.48 | b |
| 2018_19 | Crop protection | DIA | mix | 47.30 | 4.0 | 3.4 | 31.90 | 62.70 | a |
| 2018_19 | Crop protection | DIA | mono | 47.60 | 4.0 | 3.4 | 32.20 | 63.00 | a |
| 2019_20 | Crop protection | DIA | mix | 44.75 | 1.4 | 43.9 | 41.56 | 47.93 | a |
| 2019_20 | Crop protection | DIA | mono | 46.65 | 1.4 | 43.9 | 43.47 | 49.84 | a |
| 2018_19 | Crop protection | Lodging (mix\|pea mono) | mix | 0.33 | 0.1 | - | 0.22 | 0.52 | a |
| 2018_19 | Crop protection | Lodging (mix\|pea mono) | mono | 75.00 | 4.3 | - | 66.98 | 83.99 | b |
| 2019_20 | Crop protection | Lodging (mix\|pea mono) | mix | 1.58 | 0.2 | - | 1.29 | 1.94 | a |
| 2019_20 | Crop protection | Lodging (mix\|pea mono) | mono | 29.00 | 2.7 | - | 24.17 | 34.79 | b |
| 2018_19 | Crop protection | Lodging (mix\|wheat mono) | mix | 0.33 | 0.1 | 3.0 | -0.09 | 0.75 | a |
| 2018_19 | Crop protection | Lodging (mix\|wheat mono) | mono | 0.40 | 0.1 | 3.0 | -0.02 | 0.82 | a |
| 2019_20 | Crop protection | Lodging (mix\|wheat mono) | mix | 1.23 | 1.4 | 3.4 | -2.91 | 5.38 | a |
| 2019_20 | Crop protection | Lodging (mix\|wheat mono) | mono | 3.81 | 1.4 | 3.5 | -0.18 | 7.81 | a |
| 2018_19 | Yield | Pea yield | mix | 1.57 | 0.2 | 3.0 | 1.01 | 2.13 | a |
| 2018_19 | Yield | Pea yield | mono | 2.01 | 0.2 | 7.7 | 1.49 | 2.53 | b |
| 2019_20 | Yield | Pea yield | mix | 0.50 | 0.0 | 3.3 | 0.40 | 0.59 | a |
| 2019_20 | Yield | Pea yield | mono | 2.69 | 0.1 | 61.0 | 2.46 | 2.92 | b |
| 2018_19 | Yield | Total yield | mix | 4.74 | 0.2 | 3.0 | 4.07 | 5.41 | a |
| 2018_19 | Yield | Total yield | mono | 4.20 | 0.2 | 3.0 | 3.53 | 4.88 | a |
| 2019_20 | Yield | Total yield | mix | 5.78 | 0.1 | 3.0 | 5.54 | 6.03 | a |
| 2019_20 | Yield | Total yield | mono | 5.74 | 0.1 | 3.0 | 5.50 | 5.99 | a |
| 2018_19 | Crop protection | Weeds (mix\|pea mono) | mix | 1.76 | 0.1 | 3.1 | 1.48 | 2.05 | a |
| 2018_19 | Crop protection | Weeds (mix\|pea mono) | mono | 7.33 | 0.2 | 49.4 | 6.87 | 7.79 | b |
| 2019_20 | Crop protection | Weeds (mix\|pea mono) | mix | 2.34 | 0.2 | 3.2 | 1.80 | 2.87 | a |
| 2019_20 | Crop protection | Weeds (mix\|pea mono) | mono | 9.17 | 0.5 | 57.6 | 8.12 | 10.21 | b |
| 2018_19 | Crop protection | Weeds (mix\|wheat mono) | mix | 1.76 | 0.1 | 3.0 | 1.57 | 1.95 | a |
| 2018_19 | Crop protection | Weeds (mix\|wheat mono) | mono | 1.94 | 0.1 | 3.0 | 1.75 | 2.13 | a |
| 2019_20 | Crop protection | Weeds (mix\|wheat mono) | mix | 2.34 | 0.1 | 3.0 | 1.95 | 2.73 | a |
| 2019_20 | Crop protection | Weeds (mix\|wheat mono) | mono | 2.45 | 0.1 | 3.0 | 2.06 | 2.84 | a |
| 2018_19 | Quality | Wheat protein | mix | 11.99 | 0.1 | 3.0 | 11.65 | 12.33 | b |
| 2018_19 | Quality | Wheat protein | mono | 10.42 | 0.1 | 3.0 | 10.08 | 10.76 | a |
| 2019_20 | Quality | Wheat protein | mix | 13.42 | 0.1 | 3.0 | 13.18 | 13.65 | b |
| 2019_20 | Quality | Wheat protein | mono | 12.69 | 0.1 | 3.0 | 12.46 | 12.92 | a |
| 2018_19 | Quality | Wheat TGW | mix | 45.80 | 0.2 | 3.0 | 45.12 | 46.47 | a |
| 2018_19 | Quality | Wheat TGW | mono | 44.98 | 0.2 | 3.0 | 44.29 | 45.66 | a |
| 2019_20 | Quality | Wheat TGW | mix | 51.64 | 0.2 | 3.0 | 51.11 | 52.17 | b |
| 2019_20 | Quality | Wheat TGW | mono | 50.66 | 0.2 | 3.0 | 50.12 | 51.19 | a |
| 2018_19 | Yield | Wheat yield | mix | 3.17 | 0.2 | 3.0 | 2.69 | 3.66 | a |
| 2018_19 | Yield | Wheat yield | mono | 4.20 | 0.2 | 3.0 | 3.72 | 4.69 | b |
| 2019_20 | Yield | Wheat yield | mix | 5.28 | 0.1 | 3.0 | 4.99 | 5.58 | a |
| 2019_20 | Yield | Wheat yield | mono | 5.74 | 0.1 | 3.0 | 5.45 | 6.04 | b |
| 2018_19 | Crop protection | *O. yallundae* | mix | 38.4 | 3.75 | 3 | 22.8 | 54.1 | a |
| 2018_19 | Crop protection | *O. yallundae* | mono | 39 | 3.75 | 3 | 23.3 | 54.7 | a |
| 2018_19 | Crop protection | *C. cereale* | mix | 2.2538 | 0.7944 | 4.3109 | -0.4233 | 4.9308 | a |
| 2018_19 | Crop protection | *C. cereale* | mono | 3.1992 | 0.7944 | 4.3109 | 0.5221 | 5.8762 | a |
| 2018_19 | Crop protection | *Fusarium spp.* | mix | 7.7278 | 1.7835 | 4.7553 | 1.9714 | 13.4842 | a |
| 2018_19 | Crop protection | *Fusarium spp.* | mono | 5.9810 | 1.7835 | 4.7553 | 0.2246 | 11.7374 | a |
| 2019_20 | Crop protection | *O. yallundae* | mix | 41.1214 | 1.4747 | 47.8367 | 37.7087 | 44.5341 | a |
| 2019_20 | Crop protection | *O. yallundae* | mono | 43.1430 | 1.4747 | 47.8367 | 39.7303 | 46.5557 | a |
| 2019_20 | Crop protection | *C. cereale* | mix | 1.1588 | 0.4523 | 4.5990 | -0.3218 | 2.6395 | a |
| 2019_20 | Crop protection | *C. cereale* | mono | 1.4486 | 0.4523 | 4.5990 | -0.0321 | 2.9293 | a |
| 2019_20 | Crop protection | *Fusarium spp.* | mix | 2.2538 | 0.7944 | 4.3109 | -0.4233 | 4.9308 | a |
| 2019_20 | Crop protection | *Fusarium spp.* | mono | 3.1992 | 0.7944 | 4.3109 | 0.5221 | 5.8762 | a |
| 2017_18 | Yield | Wheat yield | mix | 3.3131 | 0.0842 | 3.0000 | 3.0452 | 3.5810 | a |
| 2017_18 | Yield | Wheat yield | mono | 3.2532 | 0.0842 | 3.0000 | 2.9853 | 3.5211 | a |
| 2019_20 | RE | Yield Gain | - | 0.4152 | 0.1690 | 3.3218 | -0.2425 | 1.0729 | a |
| 2018_19 | RE | Yield Gain | - | 0.7905 | 0.1690 | 3.3218 | 0.1328 | 1.4483 | b |
| 2019_20 | RE | LER | - | 1.1133 | 0.0414 | 3.3462 | 0.9529 | 1.2736 | a |
| 2018_19 | RE | LER | - | 1.5293 | 0.0414 | 3.3462 | 1.3689 | 1.6897 | b |

# Traits: days to heading and biomass

Table S 11 Anova tables wheat heading. MS: Mean square error; DF: Numerator degrees of freedom; F: F – value; P: P- value. Derived from the linear mixed effects models. Entry represents the wheat entries, cropping systems are monoculture and mixture and replicate represents the four replicate blocks in the experiment.

|  | **2018/19** |  |  |  | **2019/20** |  |  |  |
| --- | --- | --- | --- | --- | --- | --- | --- | --- |
|  | MS | DF | F | P | MS | DF | F | P |
| System (S) | 7.5 | 1 | 7.3 | **0.07** | 22.5 | 1 | 17.4 | **0.00** |
| Entry (E) | 207.8 | 14 | 199.9 | **0.00** | 351.3 | 14 | 270.7 | **0.00** |
| SxE | 3.0 | 14 | 2.9 | **0.00** | 2.1 | 14 | 1.6 | 0.09 |

Table S 12 Wheat DTH for entries. Estimated marginal means and standard error for of the seasons 2018/19 and 2019/20 from linear mixed effect models are shown. Significant differences at p<0.05 estimated from mixed models with pairwise comparison and Holm correction are shown. Significant differences are indicated by letters.

| **2018/19** | | | | **2019/20** | | | |
| --- | --- | --- | --- | --- | --- | --- | --- |
| **Entry** | **group** | **Emmean** |  | **Entry** | **group** | **Emmean** |  |
| Toborzo | H-Line | 213.5 | a | Toborzo | H-Line | 191.8 | a |
| Nemere | H-Line | 216.9 | b | Nemere | H-Line | 198.8 | b |
| Karizma | H-Line | 219.8 | c | Karizma | H-Line | 202.3 | c |
| Elit CCP | H-HP, | 220.0 | c | Elit HP | H-HP | 203.5 | c |
| Kolompos | H-Line | 223.1 | d | Kolompos | H-Line | 204.0 | c |
| OYQII | K-HP | 224.3 | de | OYQII | K-HP | 208.8 | d |
| Capo | C-Line | 225.6 | ef | Capo | C-Line | 209.5 | d |
| BSFI | K-HP | 226.0 | ef | OQII | K-HP | 209.5 | d |
| BSFII | K-HP | 226.3 | f | BSFI | K-HP | 210.0 | de |
| OQII | K-HP | 226.3 | f | BSFII | K-HP | 211.5 | e |
| Liocharls | D-HP | 228.4 | g | Liocharls | D-HP | 211.8 | e |
| Brandex | D-HP | 228.5 | g | Brandex | D-HP | 213.8 | f |
| Wiwa | C-Line | 229.3 | gh | Achat | C-Line | 214.0 | f |
| Achat | C-Line | 230.4 | h | Wiwa | C-Line | 214.3 | f |
| Butaro | C-Line | 231.0 | h | Butaro | C-Line | 215.3 | f |

Table S 13 Anova tables for wheat biomass for two seasons. MS: Mean square error; DF: Numerator degrees of freedom; F: F – value; P: P- value. Derived from the linear mixed effects models for different agronomic traits of cereal entries. Entry represents the cereal entries, the cropping system (monoculture and mixture) and replicate represents the four replicate blocks in the experiment.

|  | **2018/19** | | | | **2019/20** | | | |
| --- | --- | --- | --- | --- | --- | --- | --- | --- |
|  | MS | DF | F | P | MS | DF | F | P |
| **Wheat biomass [t ha^-1^]** | | | | | | | | |
| System (S) | 44,1 | 1 | 17,5 | **0,03** | 11,9 | 1 | 2,7 | 0,20 |
| Entry (E) | 4,2 | 14 | 1,7 | 0,08 | 30,8 | 14 | 7,1 | **0,00** |
| SxE | 2,1 | 14 | 0,8 | 0,64 | 1,5 | 14 | 0,3 | 0,99 |
| **Wheat ears [1/m2]** | | | | | | | | |
| System (S) | 85216 | 1 | 46,6 | **0,01** | 55764 | 1 | 12,7 | **0,04** |
| Entry (E) | 4269 | 14 | 2,3 | **0,01** | 15664 | 14 | 3,6 | **0,00** |
| SxE | 2788 | 14 | 1,5 | 0,12 | 4455 | 14 | 1 | 0,44 |
| **Wheat harvest index** | | | | | | | | |
| System (S) | 0,003 | 1 | 2,7 | 0,10 | 0,001 | 1 | 16,086 | 0,21 |
| Entry (E) | 0,006 | 14 | 6 | **0,00** | 0,006 | 14/87 | 123,467 | **0,00** |
| SxE | 0,001 | 14 | 1,2 | 0,31 | 0,001 | 14/87 | 2,013 | **0,03** |
| **Wheat kernels per ear** | | | | | | | | |
| System (S) | 0,1 | 1 | 0 | 0,95 | 54,5 | 1 | 4,2 | **0,04** |
| Entry (E) | 156,2 | 14 | 13,4 | **0,00** | 76,0 | 14 | 5,9 | **0,00** |
| SxE | 8,6 | 14 | 0,7 | 0,73 | 19,7 | 14 | 1,5 | 0,12 |
| **Wheat TGW [g]** | | | | | | | | |
| System (S) | 6,9 | 01-Mar | 7,3 | 0,07 | 29,0 | Jan-87 | 17,3 | **0,00** |
| Entry (E) | 147,6 | 14/82 | 156,9 | **0,00** | 74,5 | 14/87 | 44,4 | **0,00** |
| SxE | 2,4 | 14/82 | 2,6 | **0,00** | 3,8 | 14/87 | 2,2 | **0,01** |
